# *Gymnema sylvestre* saponins for potential antifungal action: *in vitro* and *in silico* perspectives

**DOI:** 10.3389/fpls.2025.1508454

**Published:** 2025-03-27

**Authors:** Shila Neel, Abhishek Mandal, Supradip Saha, Amrita Das, Aditi Kundu, Anupama Singh

**Affiliations:** ^1^ Division of Agricultural Chemicals, ICAR-Indian Agricultural Research Institute, New Delhi, India; ^2^ The Graduate School, ICAR-Indian Agricultural Research Institute, New Delhi, India; ^3^ Division of Basic Sciences, ICAR-Indian Institute of Horticultural Research, Bengaluru, India; ^4^ Division of Plant Pathology, ICAR-Indian Agricultural Research Institute, New Delhi, India

**Keywords:** phytochemicals, triterpenoids, ergosterol inhibition, phytopathogen, gymnemic acids, Gymnema saponins

## Abstract

Saponins are responsible for a wide range of biological activities, which is why the present research is focused on the chemical profiling of saponins and other metabolites from *Gymnema sylvestre* leaves for their potential efficacy in managing pathogenic fungi. Leaves of the plant was extracted with chloroform to obtain crude saponin concentrates. Characterizations of the chloroform soluble fraction of the leaves [chloroform extract of *G. sylvestre* (CGS)] in ultra-performance liquid chromatography–quadrupole time of flight–electrospray ionization–tandem mass spectrometry (UPLC-QToF-ESI-MS/MS) displayed 23 metabolites, primarily comprising of saponins and other minor phytocomponents. Among these, two major saponins, gymnemic acid IV and gymnestrogenin, were isolated, purified, and characterized using ^1^H-NMR, ^13^C-NMR, and high-resolution mass spectrometry (HRMS). *In vitro* fungistatic efficacy showed the highest effectiveness against *Penicillium digitatum* 6952 (EC_50_ 297.2 μg/mL), followed by *Penicillium expansum* 2995 (360.5 μg/mL) and *Aspergillus flavus* 6678 (369.4 μg/mL). Furthermore, the mechanism of interaction of these metabolites to inhibit cyt P_450_ sterol 1,4-α-demethylase was determined by *in vitro* and *in silico* molecular modeling analysis, explaining the probable reason for the reduction in ergosterol content in the treated fungi. *In silico* analysis suggested the highest binding efficiency of gymnemic acid IV due to the lowest binding energy, specifically interacted through conventional H-bonds, hydrophobic π-alkyl, π-π, and π-sigma interactions. Indeed, the valuable findings of the study would be useful for further development of Gymnema saponin based biopesticidal products.

## Introduction

Over the past two decades, there has been growing interest in exploring plant-derived bioactive compounds, commonly called phyto-metabolites, for their potential use in pharmaceuticals, nutraceuticals, and sustainable agriculture (Gurnani et al., 2014; Wadkar et al., 2008). These bioactive compounds, including alkaloids, flavonoids, saponins, terpenoids, and phenolics, are produced by plants as part of their defense mechanism against different phytopathogens, pests, and environmental stresses. Additionally, they offer significant potential in managing many crop diseases in field and storage, thereby improving post-harvest management practices (Muddapur et al., 2024). It was estimated that 35%–55% of crop loss occurred due to post-harvest diseases and mishandling (Pillai et al., 2024). Phyto-metabolites are found to be highly effective in controlling several notorious plant pathogens, particularly those that cause post-harvest damage to fresh produce (Srinivasan and Kumaravel, 2016). Traditionally, the management of these pathogens has relied heavily on the application of synthetic fungicides, which have proven effective but come with significant drawbacks. Continuous use of these chemical fungicides has led to issues such as pathogen resistance and the accumulation of toxic residues in food products and the environment (Devi et al., 2021; Jung et al., 2006). However, plant-based metabolites provide a promising solution, offering natural fungicidal properties with complex modes of action without the associated toxicity, resistance development, and environmental hazards (Khursheed et al., 2022).

Research on plant metabolites revealed that many of these natural products exhibit strong antifungal activity, making them viable candidates for use as biopesticides (Patil et al., 2011). Plants belonging to the Apocynaceae family include approximately 2,000 species across 250 genera, distributed across the tropical and subtropical regions (Tiwari et al., 2014; Muddapur et al., 2024). *Gymnema sylvestre*, commonly called “Gurmar” in local parlance, is a prominent herb in India, distributed widely across Konkan and the Western Ghats, as well as the Deccan peninsula and Western and Northern India (Sharma et al., 2024; Dar et al., 2024; Parveen et al., 2019). The plant contains bioactive saponins, particularly gymnemic acid derivatives, which are considered valuable compounds responsible for bioactivity. *Gymnema*-based remedies have attracted the attention of scientists due to their therapeutic efficacy in the area of alternative and complementary medicine (Romaiyan et al., 2023).

Evidence-based studies documented *G. sylvestre* phyto-molecules as powerful phytochemicals capable of combating many diseases and ailments. The leaves contain bioactive triterpenoid saponins. The primary phyto-components of the plant comprise various gymnemic acid saponins and gymnemasides (Arora and Sood, 2017; Vats et al., 2023; Rahman and Husen, 2023; Sagheer et al., 2023). Gymnemic acid analogs were extracted from *G. sylvestre* leaves and analyzed using liquid chromatographic and mass spectrometric methods (Kamble et al., 2013; Chen and Guo, 2017; Dar et al., 2024). Furthermore, a building block strategy was made to identify the oleanane triterpenoids of *G. sylvestre* using ultra-performance liquid chromatography–quadrupole time of flight–mass spectrometry (UPLC-QToF/MS) (Pham et al., 2019). Previous phytochemical investigations on different plant parts led to the isolation of certain triterpenoid saponins, flavonoids, and their glycosides, displaying a broad spectrum of biological activities (Thangavelu et al., 2012; Ahamad et al., 2018). Even the production of gymnemic acid from the suspension culture of *G. sylvestre* was investigated by Chodisetti et al. (2013). Recently, the saponin extraction process from leaves of *Gymnema* using advanced extraction techniques was optimized (Saeed et al., 2022).

Phyto-pharmacological properties of *G. sylvestre* were reported in the literature (Khan et al., 2019). In addition to antidiabetic action, the potential antimicrobial activity of the plant metabolites and their exploitation in the management of fungal organisms were also reported (Satdive et al., 2003; Ibrahim et al., 2017; Suwan et al., 2022). Significant antifungal and antibacterial activities of the saponin fractions extracted from the plant were also reported by Gopiesh Khanna and Kannabiran (2008). Again, the antiviral action of the *Gymnema* metabolites, particularly gymnemagenol, was reported in the literature (Gopiesh Khanna et al., 2011).

Extensive studies are being attempted to isolate and identify various bioactive saponins of *G. sylvestre* leaves for their ethnopharmacological and medicinal properties; however, comprehensive metabolomics profiling and characterizations of the bioactive fraction of the plant are still lacking. Furthermore, limited information is available on the antifungal action of metabolites of *G. sylvestre* such as triterpenoids, flavonoids, and steroids against the important decay-causing fungi such as *Penicillium* sp. and *Aspergillus* sp. Furthermore, a study related to the mechanism of interaction of *Gymnema* metabolites with the fungal proteins and their possible effect on fungal biology is also missing. Therefore, the present investigation emphasized the characterization of bioactive metabolites comprising saponins and other phytochemicals from *G. sylvestre* leaves for potential fungicidal action against fungi (*Penicillium* sp. and *Aspergillus* sp.) causing fungal spoilage under storage of fresh produce. Additionally, the interaction of these metabolites with the target-specific fungal protein, responsible for sterol biosynthesis, was also targeted using molecular modeling analysis.

## Material and methods

### Plant sample

Fresh leaves of *G. sylvestre* (2.0 kg) were collected from Delhi, India (28.65°N, 77.22°E) in July 2022. Leaves were cleaned with distilled water, shade-dried for 5 days, and ground into a coarse powder. The powdered sample was stored in an airtight container under refrigeration. The moisture content of the sample was 5.9% ± 0.7% (w/w).

### Chemicals and instruments

Solvents and media were procured from Merck^®^ India Ltd. (Mumbai, India) and used without further purification. Bioactive metabolites of *G. sylvestre* leaves were purified through silica gel (60–120 mesh)-loaded column chromatography sourced from Merck^®^ India Ltd. Analytical grade solvents (LC-MS grade), HSGF_254_ silica gel-coated Thin Layer Chromatography (TLC) plates, and Preparative-Thin Layer Chromatography (TLC) glass plates (0.4–0.5 mm) pre-coated with silica gel GF_254_ were also obtained from Merck^®^ India Ltd.

Nuclear magnetic resonance (NMR) spectra were obtained using a JEOL 400-MHz NMR spectrometer, with tetramethylsilane (TMS) serving as the internal standard. UPLC–QToF–electrospray ionization–tandem mass spectrometry (UPLC-QToF-ESI-MS/MS) analysis was performed on an Acquity UPLC system, connected to a quadrupole time of flight mass spectrometer (QToF-MS/MS, Xevo G2-XS system, Waters Corporation, Wilmslow, UK).

### Extraction of leaves

#### Conventional solid–liquid extraction

Phytochemical constituents from finely grounded powder (2.0 kg) of *G. sylvestre* leaves were extracted by high-speed homogenization (IKA^®^ India Private Limited, Bengaluru, India) (Dutta et al., 2021). First, the leaf powder was submerged in ethanol (20%) and homogenized for 30 min at room temperature. Then, the hydroalcoholic (20%) extract was partitioned with chloroform (1.5 L) thrice, and the CHCl_3_ soluble fraction was filtered and evaporated below 40°C using a flash evaporator (Heidolph, Schwabach, Germany) to obtain CHCl_3_ extract [chloroform extract of *G. sylvestre* (CGS)].

### Phytochemical characterizations

#### Fourier transform infrared spectroscopy

Functional groups of CGS were characterized using Fourier transform infrared spectroscopy (FTIR) (Bruker, Dresden, Germany). The FT-IR spectrum was generated by measuring the transmission percentage against the wave number ranging from 4,000 to 600 cm^−1^. For analysis, CGS (50 mg) was mixed with 100 mg of potassium bromide and pressed to prepare a pellet. The signals at the characteristic wavenumber indicated specific functional groups of the metabolites present in CGS.

#### Ultra performance liquid chromatography -quadrapole time of flight- electrospray ionization-mass (UPLC-QToF-ESI-MS)

For metabolomics analysis, a mass spectrometer (Waters Xevo-G2-XS, QToF) paired with an Acquity H Class Plus UPLC was used. The untargeted metabolomics analysis was conducted in positive electrospray ionization mode under the MS^E^ module with an m/z range of 100–1,500. QToF-ESI-MS analysis was operated using the MassLynx 4.2 software. The system captured full-scan MS data (6 V, low energy) and MS/MS data (ranging from 10.0 to 60.0 V, high energy) in the MS^E^ mode. Key parameters included a capillary voltage of 3.0 kV, a sampling cone voltage of 30 V, an extraction cone voltage of 4.5 V, a desolvation temperature of 300°C, a source temperature of 150°C, a desolvation gas flow of 800 L/h, and a cone gas flow of 30 L/h. Mass correction was achieved with a lock spray using leucine encephalin (m/z 556.2771 in positive mode) at a flow rate of 10 L/min and a concentration of 1 µg/mL every 10 seconds. Metabolites were separated using an Acquity UPLC BEH C_18_ column (2.1 mm × 100 mm, 1.8 µm) with a gradient elution system of phase A (acetonitrile with 0.1% formic acid) and phase B (water with 0.1% formic acid) at 0.3 mL/min. The gradient profile was as follows: 0–2.5 min/100% A, 2.5–4.5 min/70% A, 4.5–20.0 min/10% A, 20.0–23.0 min/5% A, and 23.0–25.0 min/100% A. The raw data were processed using the UNIFI software version 1.7, following SANTE guidelines, identifying metabolites with a mass error limit of 3 µg/mL (Munjanja, 2017; Dutta et al., 2021).

### Isolation of pure compounds

Column chromatography was employed for the separation and isolation of compounds from CGS. This technique, known for its simplicity and effectiveness, is widely used for the isolation of chemical constituents from complex mixtures. CGS (20 g) was subjected to silica gel column chromatography (60–120 mesh size, pre-activated at 110°C) using a gradient of hexane:ethyl acetate (9:1, 8:2, 7:3, 6:4, 1:1, 4:6, 3:7, 2:8, and 0:1 v/v) as the eluent, resulting in 78 fractions. Fractions 35–59, which showed three similar spots on a TLC plate, were combined and re-chromatographed using hexane:ethyl acetate (95:5 v/v) followed by preparative TLC to isolate and crystals of CGS-1 (131 mg) and CGS-2 (57 mg).

#### Gymnemic acid IV

Lemon-yellowish solid; selected ^1^H-NMR (400 MHz, CDCl_3_): δ 12.16 (1H, s, H-6″), 6.92–6.97 (1H, m, H-1′), 5.57 (1H, s, H-12), 4.49 (1H, t, J = 2.9 Hz, H-16), 4.31–4.36 (1H, m, H-3), 4.46 (1H, d, J = 4.8 Hz, H-5″), 4.52 (1H, d, J = 7.5 Hz, H-1″), 5.44 (1H, d, J = 8.6 Hz, H-21), 4.09 (1H, dd, J = 1.2, 8.2 Hz, H-2″), 4.28 (1H, s, H-28), 3.19 (1H, s, H-23), 4.23 (1H, d, J = 12.2 Hz, H-22), 4.43 (1H, dd, J = 2.5, 11.3 Hz, H-3″), 3.52 (1H, dd, J = 3.5, 11.2 Hz, H-4″), 2.17 (1H, t, J = 6.1 Hz, H-18), 2.33 (1H, d, J = 4.4 Hz, H-19), 2.21–2.26 (1H, m, H-2), 2.14 (1H, d, J = 9.2 Hz, H-11), 1.31 (1H, t, J = 3.2 Hz, H-9), 1.32–1.36 (1H, m, H-7), 1.84–1.89 (1H, m, H-6), 1.06 (3H, s, H_3_-24),1.48 (1H, d, J = 4.5 Hz, H-15), 0.74 (3H, s, H_3_-27), 1.12 (3H, s, H_3_-26), 0.64 (3H, s, H_3_-25), 1.21 (1H, t, J = 7.3 Hz, H-1), 0.87 (3H, s, H_3_-29), 0.86 (3H, s, H_3_-30). For ^13^C-NMR spectral data, see **Table 1**. The ESI-MS spectrum showed a protonated molecular ion peak at m/z 765.4424 [M + H]^+^ (C_41_H_64_O_13_).

**Table 1:** Triterpenoid saponins and phytochemical constituents of *Gymnema sylvestre* leaves (CGS) identified tentatively in UPLC-QToF-ESI-MS/MS.

Peak	R_t_ (min)	Proposed phytochemicals	Formula	Neutral mass (Da)	[M + H]^+^/[M + H]^−^	Mass error (δ, ppm)	MS/MS fragmentation pattern
1.	16.17	Gymnemoside A	C_43_H_66_O_14_	806.4452	807.4530	−2.23	788, 646, 590
2.	16.83	Mestanolone	C_20_H_32_O_2_	304.2402	305.2480	1.54	287, 259
3.	17.94	Gymnemasin A	C_47_H_74_O_17_	910.4926	911.5004	−0.76	893, 749, 587
4.	18.95	Gymnemoside B	C_43_H_66_O_14_	806.4452	807.4530	1.37	645, 483
5.	19.52	Gymnemic acid III	C41H66O13	767.4581	768.4659	0.16	591, 506, 476
6.	20.20	Uscharin	C_31_H_41_NO_8_S	587.2552	588.2591	2.23	231, 388, 569
7.	21.10	Deacylgymnemic acid	C36H58O12	682.3928	683.4011	0.73	508, 438, 368
8.	22.44	Narcissoside	C_28_H_32_O_16_	624.1690	625.1768	−0.95	463, 445, 401
9.	22.93	8-Hydroxy gymnamine	C_9_H_7_NO	145.1617	146.1695	1.67	129, 118
10.	23.83	Madecassic acid	C_30_H_48_O_6_	504.3450	505.3528	0.92	487, 469
11.	23.59	Gymnemic acid IV	C_41_H_64_O_13_	764.4346	765.4424	0.62	588, 566, 487
12.	23.76	Ursolic acid	C_30_H_48_O_3_	456.3603	457.3681	−2.37	439, 421
13.	24.10	Gymnestrogenin	C_30_H_50_O_5_	490.3658	491.3736	0.84	475, 456
14.	24.42	Lupeol	C_30_H_50_O	426.3861	427.3931	−1.87	342, 286, 258
15.	25.95	Hentriacontane	C_31_H_64_	436.5008	437.5086	0.31	421, 407, 393, 255
16.	26.06	Squalene	C_30_H_50_	410.3912	411.3990	−0.12	393, 367, 273
17.	26.69	Chelidonine	C_20_H_19_NO_5_	353.1263	354.1341	1.23	336, 321
18.	26.82	Octadecenoic acid	C18H34O2	282.2558	283.2636	−2.42	265, 239
19.	27.37	β-Amyrin	C_30_H_50_O	426.3861	427.3939	0.51	408, 398
20.	28.01	Eicosenoic acid	C_20_H_38_O_2_	310.5145	311.4584	1.93	270, 214, 286
21.	28.37	β-Sitosterol	C_29_H_50_O	414.7067	415.7145	0.64	396, 381
22.	28.78	Oleanolic acid	C_30_H_48_O_3_	456.3603	457.3681	−2.25	438, 412, 248
23.	29.02	Stigmasterol	C_29_H_48_O	412.3705	413.3775	−1.93	273, 245, 173

R_t_, retention time (min); Error (ppm), the difference between experimental mass and theoretical mass; a, positive ionization mode; UPLC-QToF-ESI-MS/MS, ultra-performance liquid chromatography–quadrupole time-of-flight–electrospray ionization–tandem mass spectrometry.

#### Gymnestrogenin

White amorphous solid; selected ^1^H NMR (400 MHz, CDCl_3_): δ 5.34 (1H, s, H-12), 4.49 (1H, t, J = 2.7 Hz, H-16), 4.73 (1H, s, H-28), 4.17 (1H, t, J = 3.4 Hz, H-3), 4.46 (1H, t, J = 5.6 Hz, H-21), 3.68 (1H, d, J = 11.2 Hz, H-22), 3.12 (1H, s, H-23), 2.72 (1H, dd, J = 3.2, 7.6 Hz, H-11), 2.43 (1H, t, J = 7.2 Hz, H 18), 2.01 (1H, d, J = 9.4 Hz, H-19), 1.86 (1H, d, J = 6.4 Hz, H-15), 1.58–1.62 (1H, m, H-2), 1.42–1.47 (1H, m, H-6), 1.55 (3H, s, H_3_-27), 1.45 (1H, t, J = 8.6 Hz, H-9), 1.14 (1H, t, J = 2.3 Hz, H-7), 1.19 (3H, s, H_3_-26), 1.15 (3H, s, H_3_-25), 0.82 (1H, t, J = 4.1 Hz, H-1), 0.82 (3H, s, H_3_-24), 0.68 (3H, s, H_3_-29), 0.57 (3H, s, H_3_-30). For ^13^C-NMR spectral data, see **Table 1**. The ESI-MS spectrum showed a protonated molecular ion peak at m/z 491.37363 [M + H]^+^ (C_30_H_50_O_5_).

### Inhibition of fungal mycelial growth

#### Pathogenic fungal strains

Pure cultures of pathogenic fungi, namely, *Aspergillus flavus* 6678, *A. flavus* 5006, *Penicillium expansum* 2995, *P. expansum* 2841, and *Penicillium digitatum* 6952, were sourced from the Indian Type Culture Collection, ICAR-Indian Agricultural Research Institute, New Delhi, India. During the assay, each fungus was sub-cultured from a respective actively growing pure culture and used for fungistatic tests.

#### Fungicidal assay

Fungistatic assessment was conducted as per the method described by Kundu et al. (2013). Briefly, the sample (50.0 mg, CGS) was dissolved in sterilized water (1.0 mL) containing 0.5% Triton X-100 emulsifier under aseptic conditions. The solution was then mixed with the sterilized molten potato dextrose agar media (50.0 mL). The CGS-infused media were poured into two sterilized Petri dishes (90.0-mm diameter) to achieve a test concentration of 1,000 μg/mL. Other test concentrations, ranging from 500 to 62.5 μg/mL, were also prepared accordingly and used against the test fungal pathogens. After allowing the media to solidify for 2 h, a mycelial disk (5-mm diameter) was cut out from the fungal colonies and inoculated at the center of the treated Petri dish. Fluconazole was used as the positive control, while sterilized distilled surfactant (0.5% Triton X-100) water acted as the negative control.

To evaluate the radial mycelial growth of the treated fungi, the colony diameter was measured until full growth was achieved on the control plate after 5–7 days depending on the fungi. The percentage of growth inhibition (I%) for each test concentration along with controls was determined. Furthermore, antifungal data were analyzed using the PoloOne software to determine the effective concentrations (EC, μg/mL) (Zulu et al., 2023).

### Inhibition of membrane ergosterol

Ergosterol was extracted from each treated fungus at different test concentrations based on a previous method with minor modifications (Kim and Lee, 2021). In brief, fungal mycelia treated with varying concentrations of CGS were subjected to extraction of membrane ergosterol. The treated mycelia were separated and combined with 10 mL of 3% methanolic KOH and stirred vigorously on a magnetic stirrer followed by heating in a water bath for 5 h. Then, the mixture was centrifuged, and the supernatant was collected. Ergosterol was then extracted by partitioning with heptane (3 × 5 mL).

Estimation of fungal ergosterol was conducted using UPLC equipped with a Photodiode Array (PDA) detector and a mass spectrometer. Separation was achieved using a C_18_ column (2.1 mm × 100 mm, 1.8 µm; Waters, Milford, MA, USA) following a gradient system for the mobile phase, consisting of ACN and H_2_O with 0.1% formic acid. The injection volume was 10 µL. The gradient program involved 0%–20% solvent A for the first 5 min, followed by 20%–100% from 5 to 18 min, and 20% solvent A from 18 to 20 min. Ergosterol was detected using the PDA detector at a λ_max_ of 282 nm. A calibration curve was generated using standard ergosterol (99% purity), and the ergosterol content in the samples was quantified. Ergosterol inhibition (%) was also calculated by comparing the ergosterol content with that of the control (untreated) (Pandey et al., 2021).

### Molecular modeling and docking

The identified metabolites of CGS were subjected to molecular docking analysis to investigate their interactions with the target protein, cytochrome P_450_ sterol 14-α-demethylase. The amino acid sequence for this fungal protein was obtained from the National Center for Biotechnology Information (NCBI) database. Additionally, the NCBI BLAST tool and Protein Data Bank (PDB) database were utilized to find appropriate templates for designing the secondary structures of the specified amino acid sequences. Subsequently, Modeller (version 9.24: r11614) was employed to model the homology protein structures, which were saved in.pdb format (Laskowski et al., 1993). The accuracy of the modeled receptor protein was evaluated using the PROCHECK software (default version Linux 64: 3.5.4). In this study, “ligands” refers to the 3D molecular structures of the identified metabolites that were used for molecular docking. The 3D structures of these compounds were prepared using Chem Draw Ultra 11.0 and saved in.sdf format. *In silico* molecular docking simulations were performed using the SeeSAR v10.3.1 software. The structure of the receptor protein was generated, and the vacant active site residues were identified. A comprehensive set of molecules was selected, generating both 3D and 2D frameworks. The interactions between the docked receptor and ligands were visualized using the Discovery Studio v4.1 visualizer (Saxena et al., 2010).

### Statistical analysis

Statistical analysis was performed using the SAS^®^ Proprietary Software version 9.4 (TS1M1), licensed to ICAR-Indian Agricultural Statistics Research Institute, New Delhi, India. Results were considered not significant when p < 0.05.

## Results

### Saponins and others bioactive metabolites

**Figure 1:**
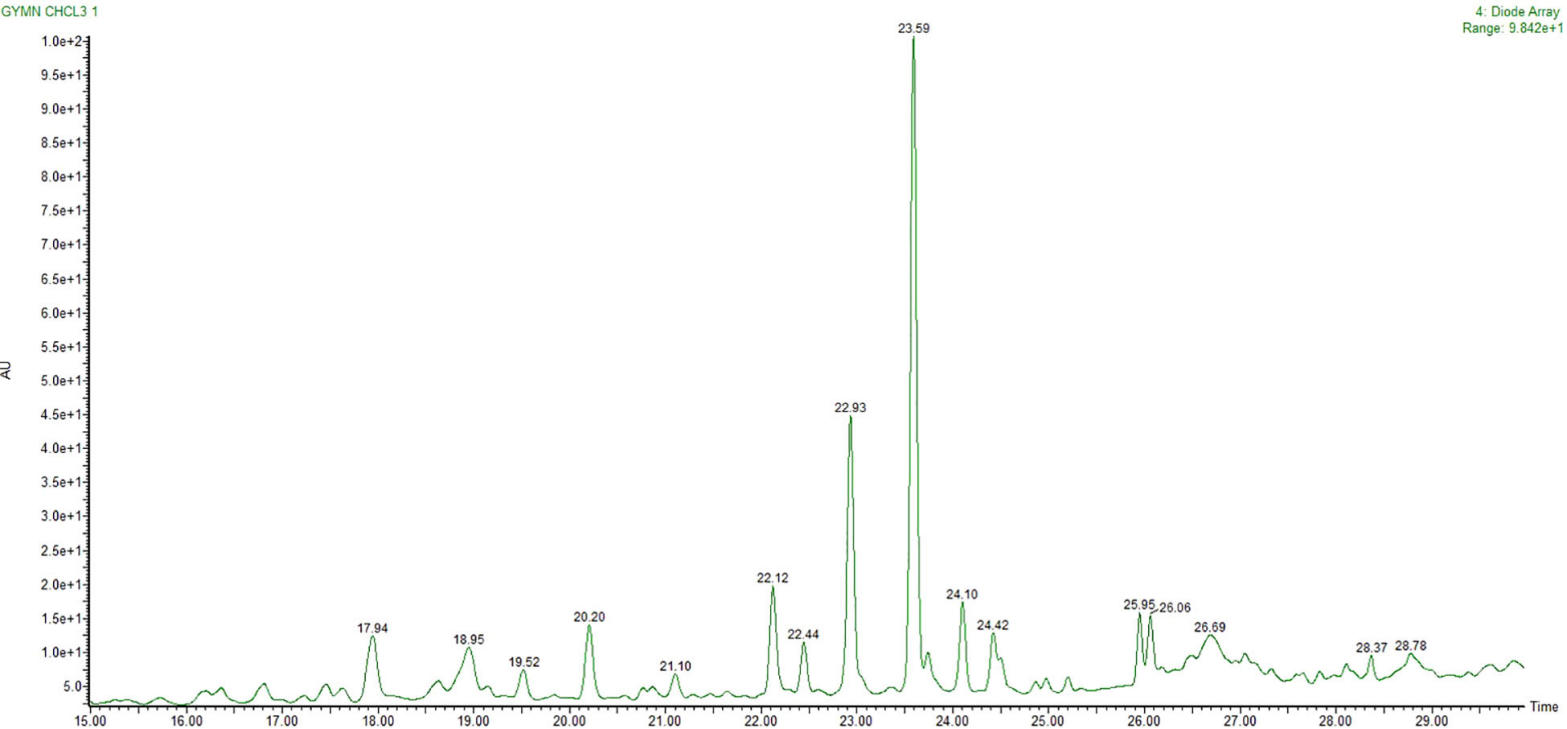
UPLC chromatogram of CGS as analyzed and separated through RP-18 column. UPLC, ultra-performance liquid chromatography; CGS, chloroform extract of *Gymnema sylvestre*.

Metabolites of CGS were determined in UPLC-QToF-ESI-MS/MS, indicating the occurrence of several major and minor components. UPLC chromatogram (**Figure 1**) of CGS displayed the detection of peaks, fully separated within 30 min of run time. Twenty-three metabolites were identified tentatively from CGS based on their exact molecular ion peaks (**Table 1**).

Gymnemoside A (C_43_H_66_O_14_), mestanolone (C_20_H_32_O_2_), gymnemasin A (C_47_H_74_O_17_), and gymnemoside B (C_43_H_66_O_14_) were eluted from the C_18_ column at the retention time (R_t_) of 16.17–18.95 min with their respective accurate mass [M + H]^+^ peaks at m/z 807.4530, 305.2480, 911.5004, and 807.4530 amu. Similarly, gymnemic acid III (C_41_H_66_O_13_), uscharin (C_31_H_41_NO_8_S), deacylgymnemic acid (C36H58O12), narcissoside (C_28_H_32_O_16_), 8-hydroxy gymnamine (C_9_H_7_NO), and madecassic acid (C_30_H_48_O_6_) were also separated and detected at the R_t_ of 19.52–23.83 min with their accurate mass [M + H]^+^ peaks at m/z 768.4659, 588.2591, 683.4011, 625.1768, 146.1695, and 505.3528 amu, respectively. Likewise, gymnemic acid IV (C_41_H_64_O_13_), ursolic acid (C_30_H_48_O_3_), gymnestrogenin (C_30_H_50_O_5_), lupeol (C_30_H_50_O), hentriacontane (C_31_H_64_), squalene (C_30_H_50_), and chelidonine (C_20_H_19_NO_5_) were also identified with their corresponding accurate mass [M + H]^+^ peaks at m/z 765.4424, 457.3681, 491.3736, 427.3931, 437.5086, 411.3990, and 354.1341 amu, respectively. Later, fatty acids, sterols, and hydrocarbons such as octadecenoic acid (C18H34O2), β-amyrin (C_30_H_50_O), eicosenoic acid (C_20_H_38_O_2_), β-sitosterol (C_29_H_50_O), oleanolic acid (C_30_H_48_O_3_), and stigmasterol (C_29_H_48_O) were also tentatively characterized from their respective accurate mass [M + H]^+^ peaks at m/z 282.2558, 426.3861, 310.5145, 414.7067, 456.3603, and 412.3705 amu, respectively.

**Figure 2:**
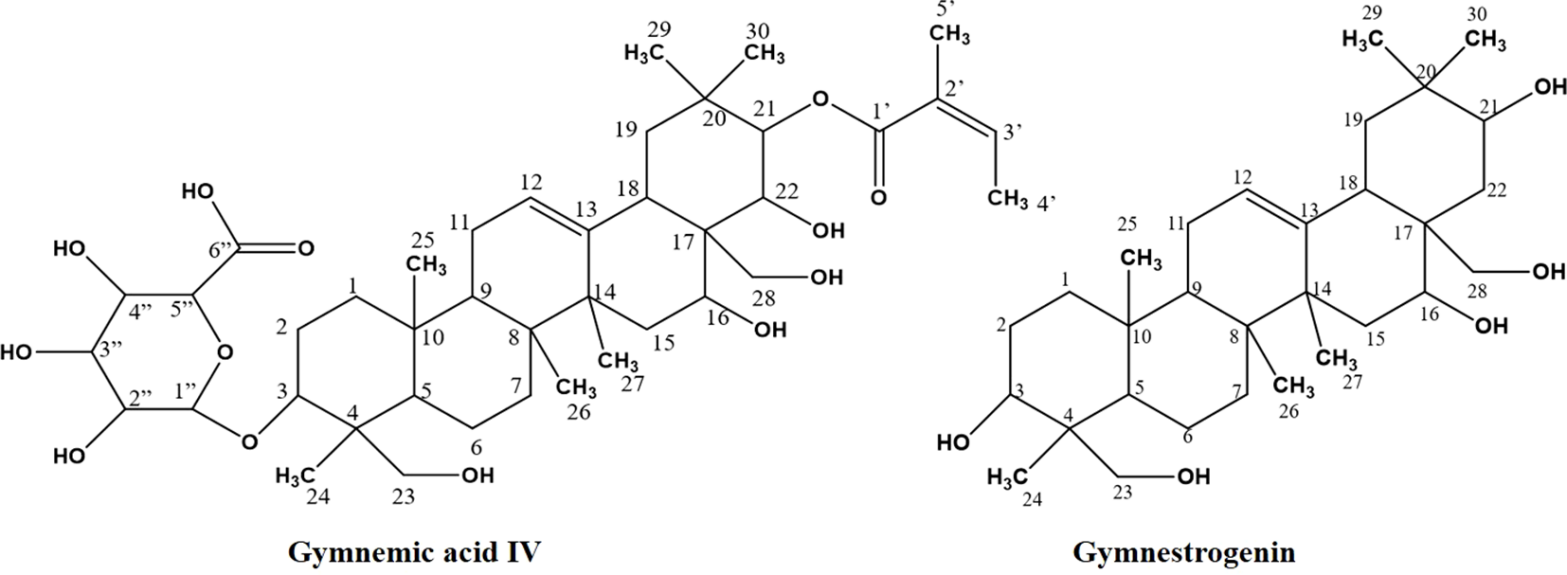
Isolation of compounds (CGS-1 and CGS-2) from chloroform extract of *Gymnema sylvestre* leaf.

**Figure 3:**
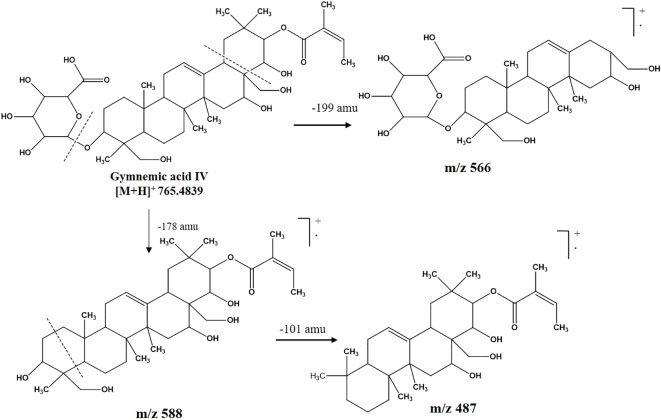
Mass fragmentation pattern of CGS-1 (gymnemic acid IV) as analyzed in UPLC-QToF-MS/MS. UPLC-QToF-MS/MS, ultra-performance liquid chromatography–quadrupole time-of-flight–tandem mass spectrometry.

Among the metabolites of CGS identified in UPLC-QToF-MS/MS, two compounds (CGS-1 and CGS-2) were isolated through column chromatography and characterized spectroscopically using ^1^H-NMR, ^13^C-NMR, and high-resolution mass spectrometry (HRMS). The purified crystals of CGS-1 appeared lemon-yellowish in color. The ^1^H-NMR spectrum of CGS-1 displayed signals as doublet at δ 5.44 ppm with the J value of 8.6 Hz and at δ 4.23 ppm with the J value of 12.2 Hz, which corresponded to the respective proton at H-21 and H-22, respectively (**Figure 2**). An additional signal as singlet appeared at δ 5.34 ppm, attributed to the proton at H-12. Other signals appeared as triplet at δ 4.49 ppm, which were assigned to the proton at H-16. Again, characteristic signals were observed in the ^13^C-NMR spectrum of CGS-1 (**Table 2**). ^13^C-NMR spectrum exhibited δ 121.8 ppm (C-12) and δ 142.4 ppm (C-13), indicating the occurrence of conjugated and aromatic carbons. The chemical shift values for the glucopyranosiduronic acid unit were evident in the spectrum, with the signals appearing at δ 106.2 ppm (C1″), δ 75.3 ppm (C2″), δ 69.7 ppm (C3″), δ 72.4 ppm (C4″), δ 76.1 ppm (C5″), and δ 172.6 ppm (C6″). Furthermore, HRMS analysis gave a sharp peak at m/z 765.4424, corresponding to its adduct [M + H]^+^ with the empirical formula C_41_H_64_O_13_ (**Figure 3**). Based on these features, CGS-1 was identified as gymnemic acid IV (**Table 2**).

**Table 2:** ^13^C-NMR spectral data for gymnemic acid IV and gymnestrogenin (δ in CDCl_3_) from the leaves of *Gymnema sylvestre*.

Position (C/H)	CGS-1		CGS-2	
	δ ^1^H (ppm)	δ ^13^C (ppm)	δ ^1^H (ppm)	δ ^13^C (ppm)
1	1.21 (t, J = 7.3 Hz)	27.9	0.82 (t, J = 4.1 Hz)	39.7
2	2.21–2.26 (m)	26.3	1.58–1.62 (m)	29.3
3	4.31–4.36 (m)	75.1	4.17 (t, J = 3.4 Hz)	72.7
4	2.49 (d, J = 2.6 Hz)	42.6	2.14 (d, J = 8.6 Hz)	44.1
5	–	52.4	–	52.8
6	1.84–1.89 (m)	17.2	1.42–1.47 (m)	19.5
7	1.32–1.36 (m)	29.7	1.14 (t, J = 2.3 Hz)	33.4
8	–	53.6	–	43.9
9	1.31 (t, J = 3.2 Hz)	48.5	1.45 (t, J = 8.6 Hz)	48.7
10	–	28.6		39.2
11	2.14 (d, J = 9.2 Hz)	24.3	2.72 (dd, J = 3.2, 7.6 Hz)	27.32
12	5.34 (s)	121.8	5.34 (s)	124.2
13	–	142.4		141.5
14	–	44.8	2.04 (s)	46.2
15	1.48 (d, J = 4.5 Hz)	34.3	1.86 (d, J = 6.4 Hz)	32.4
16	4.49 (t, J = 2.7 Hz)	64.9	5.17 (t, J = 3.7 Hz)	64.52
17	–	47.6	–	47.9
18	2.17 (t, J = 6.1 Hz)	58.1	2.43 (t, J = 7.2 Hz)	56.8
19	2.33 (d, J = 4.4 Hz)	53.6	2.01 (d, J = 9.4 Hz)	54.3
20	–	36.8	–	29.8
21	5.44 (d, J = 8.6 Hz)	78.1	4.46 (t, J = 5.6 Hz)	75.2
22	4.23 (d, J = 12.2 Hz)	74.4	3.68 (d, J = 11.2 Hz)	74.6
23	3.19 (s)	68.7	3.12 (s)	68.2
24	1.06 (s)	15.4	0.82 (s)	14.3
25	0.64 (s)	17.3	1.15 (s)	19.8
26	1.12 (s)	18.3	1.19 (s)	23.6
27	0.74 (s)	27.9	1.55 (s)	26.2
28	4.28 (s)	61.5	4.73 (s)	63.5
29	0.87 (s)	28.3	0.68 (s)	23.3
30	0.86 (s)	20.3	0.57 (s)	28.5
1’	–	171.6		
2’	1.47 (s)	131.6		
3’	6.92–6.97 (m)	146.8		
4’	1.89 (d, J = 8.9 Hz)	16.3		
5’	1.86 (s)	17.9		
1″	4.52 (d, J = 7.5 Hz)	106.2		
2″	4.09 (dd, J = 1.2, 8.2 Hz)	75.3		
3″	4.53 (dd, J = 2.5, 11.3 Hz)	69.7		
4″	3.52 (dd, J = 4.5, 9.2 Hz)	72.4		
5″	4.46 (d, J = 4.8 Hz)	76.1		
6″	12.16 (s)	172.6		

s, singlet; d,  doublet; t,  triplet; m, multiplet; dd, double doublet; br, broad; J,  coupling constant; Hz, hertz.

**Figure 4:**
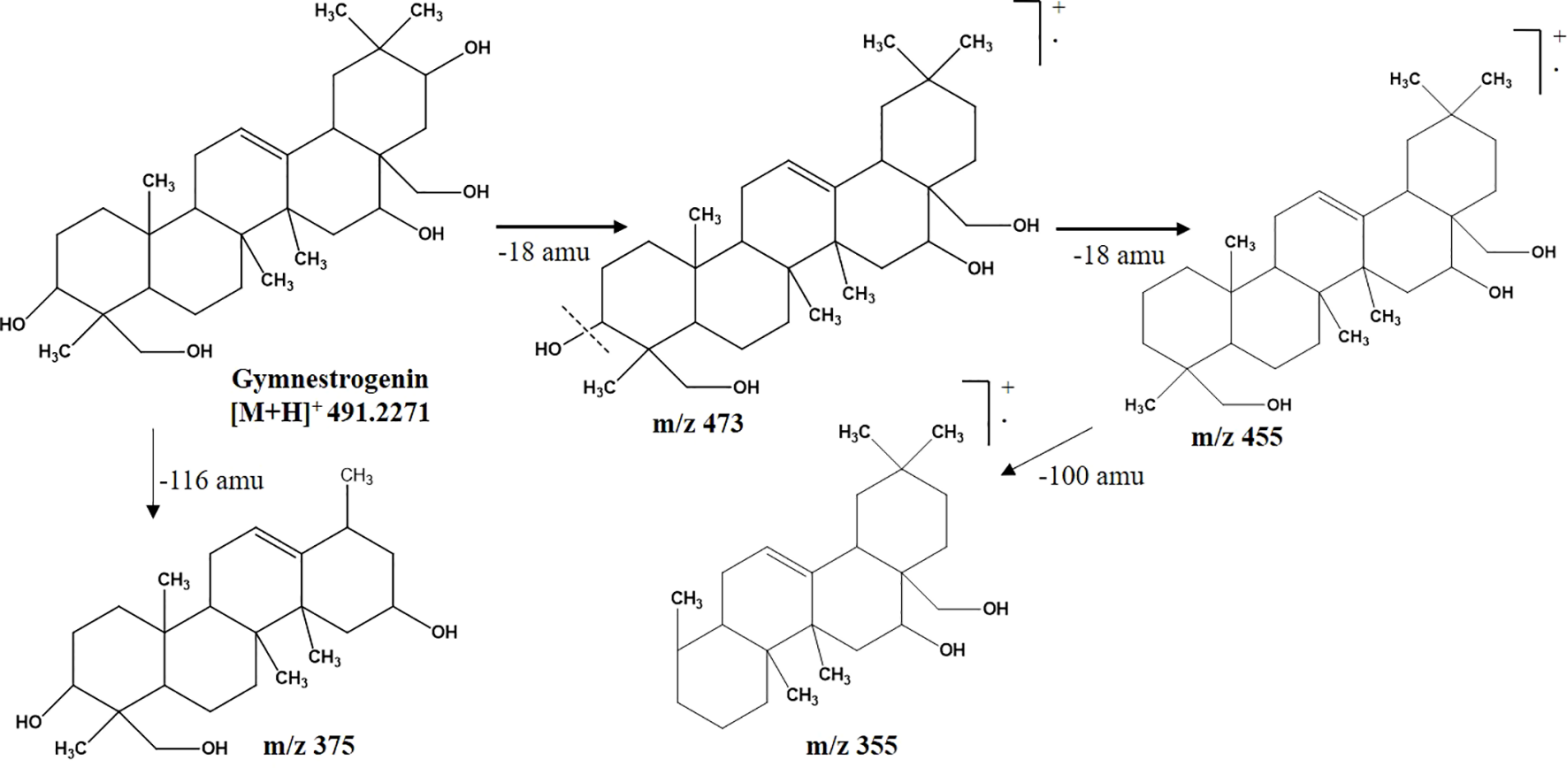
Mass fragmentation pattern of CGS-2 (gymnestrogenin) as analyzed in UPLC-QToF-MS/MS. UPLC-QToF-MS/MS, ultra-performance liquid chromatography–quadrupole time-of-flight–tandem mass spectrometry.

CGS-2 was also purified from CGS using column chromatography and recrystallized, which appeared as a white amorphous crystal. The ^1^H-NMR spectrum of CGS-2 gave a signal as singlet at δ 5.34 ppm, attributed to the H-12 proton. Other signals were detected as triplet at δ 4.46 ppm with a J value of 5.6 Hz and at δ 3.68 ppm as doublet with a J value of 11.2 Hz, corresponding to the H-21 and H-22 protons, respectively. Another signal appeared as singlet at δ 4.73 ppm, attributed to the proton at H-28 of CGS-2. Similarly, the ^13^C-NMR spectrum of CGS-2 exhibited signals at δ 124.2 ppm (C-12) and δ 141.5 ppm (C-13), suggesting the presence of allylic and aromatic carbons. Carbons at δ 52.6 ppm (C-5) and δ 56.8 ppm (C-18) reflected the presence of aliphatic hydrocarbons. Additional signals at δ 75.2 ppm (C-21) and δ 74.6 ppm (C-22) corresponded to the carbon attached with hydroxyl functionalities. HRMS analysis of the purified molecule (CGS-2) gave [M + H]^+^ peak at m/z 491.37363 with the corresponding empirical formula C_30_H_50_O_5_ (**Figure 3**). With these characteristic features, the molecule CGS-2 was identified as gymnestrogenin (**Figure 4**).

### Fungal growth inhibition

The antifungal effectiveness of CGS against selected fungal pathogens such as *A. flavus* 6678, *A. flavus* 5006, *P. expansum* 2995, *P. expansum* 2841, and *P. digitatum* 6952 revealed broad-spectrum activity. Among the tested fungi, strains of *P. digitatum* 6952 were highly susceptible to CGS, exhibiting more than 50% mycelial growth inhibition. Maximum growth inhibition (%) was recorded against *P. digitatum* 6952, *P. expansum* 2995, and *A. flavus* 6678. Additionally, significant growth inhibition was also noticed against *P. expansum* 2841 and *A. flavus* 5006. Antifungal action of the bioactive saponin-rich CGS showed a higher response in terms of activity, which increased with the concentration. Fungistatic efficacy of CGS showed EC_50_ 297.2, 360.5, and 369.4 μg/mL against *P. digitatum* 6952, *P. expansum* 2995, and *A. flavus* 6678, respectively (**Table 3**). The order of fungistatic action followed *P. digitatum* 6952 > *P. expansum* 2995 > *A. flavus* 6678 > *P. expansum* 2841 > *A. flavus* 5006. However, fluconazole (positive control) was more inhibitory against *P. digitatum* 6952 and *P. expansum* 2995 with EC_50_ 67.3 and 79.0 μg/mL, respectively.

**Table 3:** Growth inhibition (EC_50_, µg/mL) of five different fungal strains of storage pathogens treated with chloroform extract of *Gymnema sylvestre* leaves.

Pathogen	^a^EC_50_ (µg/mL)	95% Confidence limit (µg/mL)		^b^Slope ± SE	^c^Intercept ± SE	^d^(χ^2^)
		Lower	Upper			
*Aspergillus flavus* 6678	369.4	279.1	517.6	1.59 ± 0.015	−0.57 ± 0.23	5.40
*A. flavus* 5006	433.9	341.4	584.9	1.14 ± 0.14	−0.57 ± 0.20	1.30
*Penicillium digitatum* 6952	297.2	212.3	369.8	1.14 ± 0.14	−0.48 ± 0.20	0.50
*Penicillium expansum* 2995	360.5	288.5	465.7	1.18 ± 0.14	−0.50 ± 0.20	1.73
*Penicillium expansum* 2841	427.6	284.2	785.0	1.22 ± 0.14	−0.52 ± 0.21	3.93

a EC_50_, effective concentration (µg/mL) at which 50% mycelial growth inhibition recorded.

b Slope response of regression equation ± standard error.

c Intercept regression equation ± standard error.

d χ^2^, chi-squared value.

Furthermore, the influence of the bioactive metabolites (saponins) of CGS on the fungal species was also assessed by determining the membrane ergosterol content of the treated fungal pathogens. Ergosterol is a functional sterol that generally imparts strength to the fungal membrane structure, providing stability and integrity. In this context, the efficacy of CGS was determined using UPLC-MS, which indicated complete inhibition of ergosterol content at 2,000 µg/mL concentration of CGS. More than 50% inhibition in ergosterol content (57.61% ± 2.08%) was recorded in *P. digitatum* 6952 at the concentration of 125 µg/mL (**Table 4**).

**Table 4:** Ergosterol inhibition (%) with the treatment of chloroform soluble fraction at different concentrations against selected fungal pathogens.

Conc. (µg/mL)	Ergosterol inhibition ^*^(%)				
	*Penicillium digitatum* 6952	*Penicillium expansum* 2995	*Aspergillus flavus* 6678	*P. expansum* 2841	*A. flavus* 5006
62.5	39.35 ± 2.74	35.85 ± 1.24	22.72 ± 2.53	28.84 ± 2.09	31.01 ± 3.48
125	59.61 ± 2.08	57.38 ± 3.13	31.62 ± 3.69	45.31 ± 1.76	53.74 ± 2.61
250	66.73 ± 2.18	64.77 ± 2.74	58.50 ± 2.61	54.36 ± 2.69	60.81 ± 1.87
500	82.99 ± 2.42	79.92 ± 3.02	69.33 ± 2.42	68.18 ± 3.86	65.46 ± 2.27
1,000	89.43 ± 3.96	87.31 ± 2.65	80.32 ± 1.81	76.19 ± 2.31	69.28 ± 1.97
2,000	100 ± 0.00	100 ± 0.00	100 ± 0.00	100 ± 0.00	100 ± 0.00

^*^Results are shown as mean ± SD.

### Interaction with target protein

**Figure 5:**
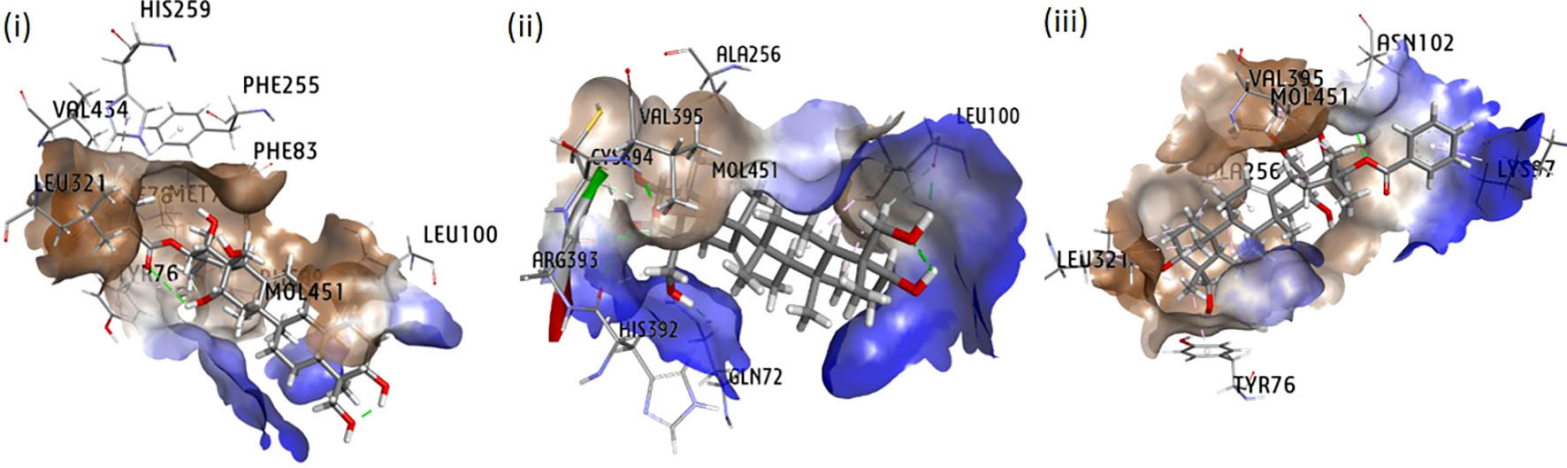
Interaction in 3D poses of *Gymnema sylvestre* metabolites with protein squalene epoxidase (i) gymnemic acid IV, (ii) gymnemoside A, and (iii) gymnestrogenin.

Molecular docking analysis was extensively exploited recently to explain the functional interaction of the metabolites with target-specific enzymes, facilitating possible inhibition of the enzymes. Metabolites identified in CGS were used for the analysis of their potential inhibitory action toward cyt P_450_ sterol 1,4-α-demethylase. The results revealed that the saponins exhibited promising docking scores ranging from −21.7 to −6.3 kcal/mol (**Table 5**). Based on the poses generated through molecular docking, the potential molecules engaging in protein–ligand complexes are mentioned in decreasing order: gymnemic acid IV > gymnemoside A > gymnestrogenin > gymnemic acid III > gymnemoside B > gymnemasin A > lupeol. The lower binding affinity range indicated favorable interactions between the molecules (ligands) and cyt P_450_ sterol 1,4-α-demethylase (target protein). Among the tested compounds, gymnemic acid IV was identified as the most efficient binder with the lowest binding energy (–21.7 kJ/mol), suggesting better stability of the ligand–protein complex. Furthermore, the ligand efficiency (LE) and lipophilic ligand efficiency (LLE) were also deduced to determine their ability to bind the sterol biosynthesizing protein based on their respective lipophilic property. Herein, the steroidal and triterpenic structures of the saponins may have helped to make the strong ligand–protein complex, facilitating the possibility of blocking the target protein. Based on the binding affinity and associated free energy, the promising molecules are mentioned as gymnemic acid IV (−21.7 kJ/mol), gymnemoside A (−19.5 kJ/mol), and gymnestrogenin (−16.5 kJ/mol). Furthermore, gymnemic acid IV showed interactions with specific amino acid residues such as HIS259, VAL434, PHE255, PHE83, LEU321, MOL451, and LEU100 at the target sites of the enzyme (**Figure 5**). The stability of the ligand–protein complex could be attributed to low-distance conventional hydrogen bonds and hydrophobic pi-alkyl interactions.

**Table 5:** Molecular docking scores and energies associated with the enzyme complex containing major components of *Gymnema sylvestre* leaves.

Identified compounds	Log P	Binding affinity range (nM)	ΔG	LE	LLE
Gymnemic acid IV	2.30	19,560.06 < KI < 1,943,407.61	−21.7	–	–
Gymnemoside A	3.02	40,371.02 < KI < 4,011,099.55	−19.5	–	–
Gymnestrogenin	4.13	134,723.46 < KI < 13,642,372.71	−16.5	–	–
Gymnemic acid III	2.01	15,920.64 < KI < 1,581,810.32	−15.1	–	–
Gymnemoside B	4.54	3,751,403.48 < KI < 372,724,116.5	−8.1	–	–
Gymnemasin A	1.71	1,013,571.01 < KI < 100,704,272.7	−6.3	–	–
Lupeol	2.53	2,332,832.65 < KI < 136,148,241.2	−2.4	–	–

LE, ligand efficiency; LLE, lipophilic ligand efficiency.

## Discussion

Metabolomics profiling and analysis of *G. sylvestre* leaves have shown the dominant composition of triterpenoid saponins, along with other phyto-molecules. Saponins of the plant have been studied extensively through either comprehensive profiling or stepwise isolation, purification, and characterization techniques (Alkefai et al., 2019). In the current investigation, saponins with diverse structural components along with other minor metabolites have been identified using chromatographic and spectroscopic techniques. UPLC-QToF-ESI-MS/MS-based tentative characterizations following mass accuracy along with fragmentation patterns indicated the identification of 23 metabolites, including gymnemic acid IV (765.4424), deacylgymnemic acid (683.4011), gymnestrogenin (491.3736), and hentriacontane (437.5086).

Metabolomics investigation of the methanolic extract of *G. sylvestre* stem bark has revealed ample triterpenoids, sterols, and flavonoids (Ditchou et al., 2024; Neel et al., 2025). Similarly, the presence of arylated gymnemic acids and *Gymnema* saponins has been confirmed in the aqueous extract of *G. sylvestre* leaves (Alkefai et al., 2018). Likewise, two triterpenoid saponins, namely, gymnemosides W_1_ and W_2_, have been isolated and characterized from the plant (Zhu et al., 2008). In the present study, triterpenoid saponins have been primarily characterized along with other constituents. Furthermore, gymnemic acid IV and gymnestrogenin have also been isolated and characterized from the saponin-rich extracts using chromatographic and spectroscopic techniques. These findings have been consistent with those reported by Renga et al. (2015), suggesting the dominant composition of oleanane saponins in *G. sylvestre*.

CGS has been found to be highly effective against storage fungi, which could be attributed to the biofunctional metabolites, mainly gymnemic acid derivatives, particularly gymnemic acid IV, gymnemoside A, and gymnestrogenin. Saponins and other valuable metabolites of the plant have been recognized for multidimensional biofunctional properties. Even the semi-nonpolar extracts (CHCl_3_ soluble fraction) of *G. sylvestre* aerial parts have been found effective for antimicrobial activity as compared to the other solvent extracts (Chodisetti et al., 2013), indicating the presence of the most prominent components in the CHCl_3_ soluble fraction.

In the current investigation, the fungistatic action of *G. sylvestre* leaves has been demonstrated against *P. digitatum* 6952, *P. expansum* 2995, *A. flavus* 6678, *P. expansum* 2841, and *A. flavus* 5006. Herein, *Gymnema* extract has been tested against the two different strains of *P. expansum* along with different species and other fungi. Previous reports suggested that variable responses of the different strains of the same species could be due to their difference in the virulence level (Kundu et al., 2022; Neel et al., 2023). In the current study, treatment of CGS has been found to be the most effective in arresting the growth of *P. digitatum* 6952, exhibiting EC_50_ 297.2 µg/mL. Furthermore, CGS was also inhibitory to the other pathogens, indicating broad-spectrum activity. Future research has been planned to utilize the most bioactive *Gymnema* chloroform fraction for the development of suitable coating formulation to make the process economical. Nevertheless, the purified compounds gymnemic acid IV and gymnestrogenin have not been further tested against the fungi. However, there could be a possibility of better antifungal action with these isolated compounds. In line with our study, ethanolic and ethyl acetate extracts of the plant have been reported to possess antimicrobial activity against various pathogenic bacteria (Satdive et al., 2003; Arora and Sood, 2017). As far as our literature survey could ascertain, the potential of chloroform soluble fraction of *G. sylvestre* to control fungal pathogens has not been reported previously. Thus, the present research could be considered the first report on the fungistatic action of the saponins of *G. sylvestre* leaves against spoilage-causing fungal pathogens.

In order to explain the efficacy of *Gymnema* metabolites in arresting fungal growth, membrane ergosterol content in the treated fungi has been studied. Ergosterol is an important component of fungal membranes, providing integrity and strength to the membrane. Hampering ergosterol biosynthesis could lead to less deposition on the membrane, hence the possibility of disintegration of fungal membrane damage (Kumar et al., 2023). In the present study, complete inhibition of ergosterol production has been noticed in the fungi at 0.2% concentration of CGS. Notably, the treated fungal pathogens displayed a significant reduction in membrane ergosterol content with the saponin-rich metabolites of *G. sylvestre* leaves; therefore, membrane integrity has been severely compromised. Inhibition of ergosterol production in the treated fungi has been consistent; hence, these findings have also been confirmed through an *in silico* analysis.

*In silico* molecular modeling of *Gymnema* metabolites to inhibit/block the target-specific protein, cyt P_450_ sterol 1,4-α-demethylase, responsible for ergosterol production in fungi, has been explained, suggesting positive interaction of the metabolites over the native ligand. In order to decipher the probable mechanism of inhibition by these molecules, stable conformations of these metabolites forming low energy bonds within the amino acid residues at the specific binding pocket of the protein have been studied. Strong interactions between amino acid residues and metabolites were confirmed through the docking scores and corresponding associated binding energies. Major metabolites have been found to be energetically favorable and qualitatively aligned with the *in vitro* analysis. Discrete attempts have been made to understand the possibilities of triterpenoids from *G. sylvestre* to inhibit α-glycosidase (Parveen et al., 2019).

Ligand–receptor-based molecular docking analysis of the identified metabolites of *G. sylvestre* leaves with the fungal ergosterol biosynthesizing enzyme cyt P_450_ sterol 1,4-α-demethylase has been found to be significant enough to consider the disruption of fungal ergosterol production, thereby damaging fungal membrane. The interaction complex of the compound gymnemic acid IV with the target protein has been found to be highly stable. Furthermore, multiple amino acid residues at site 1 of the enzyme have been identified in the interaction, forming a thermodynamically stable complex. Herein, gymnemic acid IV has been found to be the most effective with the lowest binding energy and desired LE and LLE parameters to block the enzyme with conventional H-bonds, hydrophobic π-alkyl, π-π, and π-sigma interactions. Additionally, gymnemoside A and gymnestrogenin have also been recognized next in order with respect to binding energy and stability to inhibit the protein. The interaction of gymnemic acid IV, gymnemoside A, and gymnestrogenin with cyt P_450_ sterol 1,4-α-demethylase for the first time has been reported to predict the inhibition mechanism of such saponins from *G. sylvestre* leaves.

## Conclusion

In summary, comprehensive profiling and characterizations of metabolites of *G. sylvestre* leaves have been conducted to identify 23 metabolites that showed significant fungistatic action to inhibit the growth of fungal pathogens *P. digitatum* 6952, *P. expansum* 2995, *A. flavus* 6678, *P. expansum* 2841, and *A. flavus* 5006, responsible for causing rot during post-harvest operations of agricultural produce. The saponin-rich fraction comprising abundant gymnemic acid IV along with other metabolites has been found to be highly inhibitory toward the fungi and also affects their ergosterol production. Molecular docking analysis revealed enough evidence regarding the possible interactions of these metabolites with the ergosterol biosynthesizing fungal protein, which could be responsible for the inhibition of ergosterol production in the treated fungi. These encouraging scientific findings could be of high importance for the exploitation of *G. sylvestre* under sustainable product development in crop protection.

## Data Availability

The original contributions presented in the study are included in the article/supplementary material. Further inquiries can be directed to the corresponding author.
